# Relationship between plaque composition by virtual histology intravascular ultrasound and clinical outcomes after percutaneous coronary intervention in saphenous vein graft disease patients: study protocol of a prospective cohort study

**DOI:** 10.1186/s12872-018-0975-1

**Published:** 2018-12-12

**Authors:** Yin Liu, Hai-Bo Wang, Xiang Li, Jian-Yong Xiao, Ji-Xiang Wang, Kathleen H. Reilly, Bo Sun, Jing Gao

**Affiliations:** 1 Department of Cardiology, Tianjin Chest Hospital, No.261 Tai er zhuang Road, Jinnan District Tianjin, 300222 People’s Republic of China; 2 Peking University Clinical Research Institute, Xueyuan Rd 38#, Haidian Dist, Beijing, 100191 People’s Republic of China; 3 Independent Consultant, New York City, NY USA; 4 Cardiovascular Institute, Tianjin Chest Hospital, No.261 Tai er zhuang Road, Jinnan District Tianjin, 300222 People’s Republic of China

**Keywords:** Saphenous vein graft disease, Virtual histology intravascular ultrasound, Percutaneous coronary intervention, Major adverse cardiac events

## Abstract

**Background:**

Plaque composition and morphologic characteristics identified by virtual histology intravascular ultrasound (VH-IVUS) can determine plaques at increased risk of clinical events following percutaneous coronary intervention (PCI) among coronary artery disease (CAD) patients. However, there have been few studies to investigate the relationship between plaque composition of saphenous vein graft (SVG) by VH-IVUS and clinical outcomes in patients with saphenous vein graft disease (SVGD) undergoing PCI. The purpose of this study is to determine whether plaque components and characteristics by VH-IVUS can predict major adverse cardiac events (MACEs) among SVGD patients undergoing PCI.

**Methods/design:**

This is a prospective cohort study conducted in Tianjin Chest Hospital, China. Participants with SVGD referred for PCI will be invited to participate in this study, and will be followed up at 1, 6, 12, 24 and 36 months post-PCI to assess clinical outcomes.

The planned sample size is 175 subjects. We will recruit subjects with SVGD scheduled to receive PCI, aged 18–80 years, with a history of previous coronary artery bypass graft (CABG) surgery more than 1 year ago, and willing to participate in the study and sign informed consent.

The composite primary study endpoint is the incidence of MACEs after PCI for SVGD, including death from cardiac causes, non-fatal myocardial infarction, unplanned target lesion revascularization (TLR) and target vessel revascularization (TVR). The primary outcome analysis will be presented as Kaplan-Meier estimates and the primary outcome analysis will be carried out using a Cox proportional hazards regression model.

**Discussion:**

Once the predictive values of plaque components and characteristics by VH-IVUS on subsequent clinical outcomes are determined among SVGD patients undergoing PCI, an innovative prediction tool of clinical outcomes for SVGD patients undergoing PCI will be created, which may lead to the development of new methods of risk stratification and intervention guidance.

**Trial registration:**

The study is registered to ClinicalTrials.gov (NCT03175952).

## Background

Coronary artery bypass graft (CABG) surgery is a widely used surgical procedure to treat coronary artery disease (CAD). Saphenous vein grafts (SVGs) are commonly used in CABG due to the advantage of availability, although the patency rates of SVGs are lower than that for arterial grafts [1–3]. However, about 40–50% of the SVGs will be occluded within 10 to 15 years after CABG surgery [1, 4, 5]. Newly developed atherosclerosis is the major reason for long-term poor prognosis [3]. SVG disease (SVGD), defined as a stenosis of 50% or more of the SVGs excluding distal anastomotic occlusion, has become an important cause of morbidity and mortality for CAD patients after CABG surgery [6, 7].

Both repeat CABG and percutaneous coronary intervention (PCI) are available treatment options for the management of SVGD [8–10]. Due to increased morbidity and mortality associated with patients undergoing repeat CABG, SVG PCI is the preferred therapeutic option to restore vessel patency and improve symptoms for patients with SVGD [11–13]. PCI in patients with prior CABG comprises up to 6% of the total PCI performed in the United States [14]. However, because of accelerated intimal proliferation and hyperplasia in venous conduits, SVG PCI is associated with increased risk of late failure and worse outcomes compared with native coronary artery interventions [15, 16]. Previous studies have shown that the incidence of major adverse cardiac events (MACEs) in patients who undergo bypass-graft PCI is significant higher than that in patients with native coronary artery PCI [17, 18]. It would be of particular interest to identify patients with poor prognosis after SVG PCI, which can be used for risk stratification and intervention guidance.

In recent years, intravascular ultrasound (IVUS) has been developed to identify vulnerable atherosclerotic plaques at high risk for coronary events. It was found that plaque characteristics (i.e., plaque burden, multiple plaque ruptures, lipid pool-like image and minimum luminal area) may be associated with no reflow phenomenon after PCI [19–22]. However, IVUS imposes limitations on identifying specific plaque components. Virtual histology-IVUS (VH-IVUS) is most widely used as it can assess both plaque composition and morphologic characteristics. VH-IVUS utilizes spectral and amplitude analysis of IVUS radiofrequency data to characterize plaque components, also suggesting the potential to identify vulnerable lesions [23, 24]. Moreover, several studies have demonstrated the independent relationship between VH-IVUS–defined plaque classification or plaque composition and MACEs [25, 26].

Considering similar pathophysiological mechanisms for CAD and SVGD, we hypothesized that plaque characteristics of SVG assessed by VH-IVUS might be associated with long-term clinical outcomes. However, data on plaque composition of SVG and its predictive values for clinical outcomes are still limited [27]. Thus, we sought to investigate the relationship between plaque composition of SVG by VH-IVUS and clinical outcome in SVGD patients undergoing PCI, which could identify morphologic features that are predictive of post-PCI MACEs.

## Methods and design

### Study design and setting

**Fig. 1:**
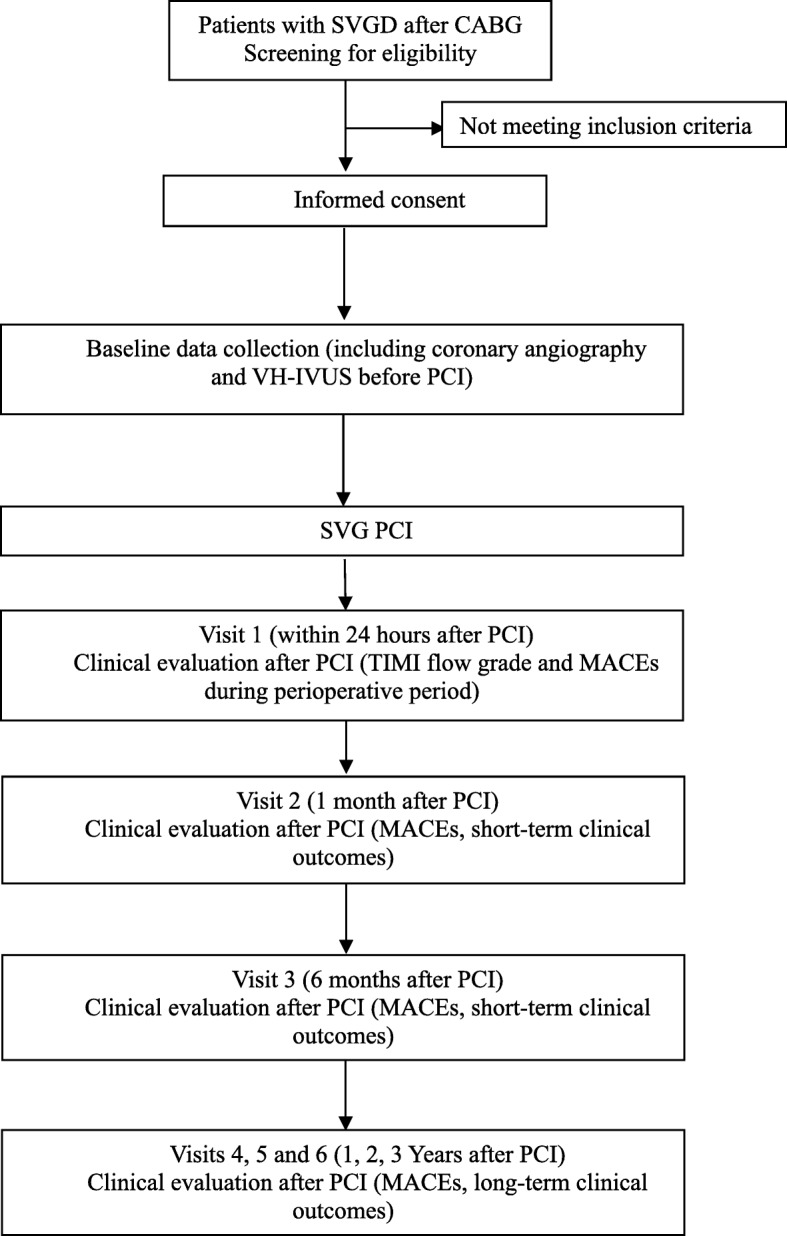
Study flow chart

The study is a prospective cohort study conducted in Tianjin Chest Hospital, Tianjin city, China. Patients with SVGD referred for PCI are invited to participate in the study and VH-IVUS is performed in culprit SVG before and after PCI. Patients are recruited consecutively by surgeons at the Cardiology Department of Tianjin Chest Hospital. These subjects will be followed up at 1, 6, 12, 24 and 36 months post-PCI to assess clinical outcomes. The flow diagram of the study and the detailed study procedures are illustrated in Fig. 1.

### Participants

The patients to be included in the study should meet all of the following criteria: (1) aged 18–80 years; (2) a history of previous CABG surgery more than 1 year ago; (3) diagnosed as SVGD which is defined as at least one SVG ≥50% diameter stenosis; (4) plans for receiving PCI using drug-eluting stents; (5) willing to participate in the study and sign informed consent.

Patients will be excluded from the study if any of the following criteria is met: (1) acute myocardial infarction within the previous 7 days; (2) having any contraindication to aspirin, clopidogrel, heparin or stainless steel; (3) impaired renal or hepatic function; (4) history of cerebral stroke or ischemic cerebrovascular disease within 3 months; (5) history of gastrointestinal bleeding or hemoptysis in the previous 4 weeks; (6) pregnant or breastfeeding; (7) vasculitis or other non-atherosclerotic CAD; (8) other major illnesses that would expose the participant to unexpected risk: hematologic disorder, malignancy, etc.

Participants can withdraw from the study at any time without any adverse outcome on subsequent treatment; the reasons for withdrawal will be recorded. The investigator can also exclude a participant from the study for reasons including serious adverse events or poor compliance with research protocol.

### Evaluation of the coronary angiography

Coronary angiography is performed using angiography system with a flat-panel detector (Philips Allura Xper FD10, Philips Healthcare, Netherlands) according to the Judkins technique. Angiography of the in situ coronary artery is performed with 6F catheters through radial or femoral access, while Judkins R (JR) 4.0 is used as the first choice catheters for SVG angiography. The location of SVG is roughly determined according to previous CABG surgery procedure and it is displayed in at least two different projections. The presence of significant stenosis (≥50%) in at least one SVG is defined as SVGD.

### VH-IVUS examination and plaque classification

After administration of heparin (100 U/Kg) and glyceryl trinitrate (100–200 μg), VH-IVUS is performed in SVG and other main coronary arteries before and after PCI. Data is obtained using a 20-MHz, 3.5-French EagleEye phased-array Gold catheter (Volcano Corporation, Rancho Cordova, California, USA) with motorized pullback (0.5 mm per second) from the most distal safe position to guide the catheter. The IVUS grey-scale and Virtual histology analyses are performed offline using the Volcano Image Analysis Software (version 3.0.394,Volcano Corporation, USA) and are not used for PCI guidance or subsequent management. Spectral and amplitude analyses of IVUS backscattered radiofrequency are performed by two experienced interventional cardiologists without knowledge of subsequent clinical events. Angiographic qualitative assessment and quantitative measurements are obtained for SVG. Quantitative IVUS measurements include cross-sectional areas (CAS) of the lumen, the external elastic membrane (EEM), and the plaque and media (EEM CAS minus lumen CAS), lesion length, plaque burden (plaque and media CAS divided by EEM CAS), and plaque volume. Qualitative IVUS assessments include plaque rupture (intraplaque cavity that communicated with the lumen with an overlying residual fibrous cap fragment) and characterization of lesions. On the basis of radiofrequency IVUS, plaque components and morphology are classified into the following four types: fibrous tissue (FT), fibrofatty (FF), necrotic core (NC), and dense calcium (DC). A lesion on IVUS imaging is defined as a plaque burden ≥40% in at least 3 consecutive frames. Consistent with published VH-IVUS classifications [25, 28], such lesions are classified as one of the following: 1) fibrotic plaque, mainly fibrous tissue with < 10% confluent NC, < 10% confluent DC, and < 15% FF; 2) fibrocalcific plaque (FCa), mainly fibrous tissue with > 10% confluent DC but < 10% confluent NC; 3) pathologic intimal thickening (PIT), not meeting FCa plaque definitions and predominantly fibrous tissue; 4) fibroatheroma, > 10% confluent NC, including thick-cap fibroatheroma (ThCFA) and thin-cap fibroatheroma (TCFA).

### Baseline assessment and follow-up

After receiving written informed consent, clinical staff will collect demographic and clinical characteristics from all participants. Medical records will be reviewed and related clinical information will be extracted. The data to be collected at baseline include demographic characteristics, medical history (diabetes mellitus, hypertension, hyperlipaemia, stroke, arrhythmia, previous myocardial infarction, PCI or CABG surgery, and medicines for cardiovascular disease), physical examination, clinical presentation (vital signs at hospital admission, onset time of chest pain, duration of chest pain, cardiac function with Killip classification), and smoking history. Cardiac color ultrasound, coronary angiogram, pre- and post-PCI VH-IVUS and 18 lead ECG will be done and used for checking inclusion/exclusion criteria. In addition, blood samples will be collected for laboratory testing, including routine blood, blood biochemistry, fasting glucose, coagulation function, homocysteine, cardiac damage markers (lactic dehydrogenase, creatine kinase, B-type natriuretic peptide, hydroxybutyrate dehydrogenase, troponin H, myoglobin), and inflammatory factors.

Eligible participants will be hospitalized for PCI treatment. In the therapy phase, patients will receive PCI for SVGD using drug-eluting stents. Operation notes (operation date, bleeding volume, medication), thrombolysis in myocardial infarction (TIMI) flow, corrected TIMI frame count (CTFC) and adverse events will be recorded in detail.

All subjects will be followed up at 1, 6, 12, 24 and 36 months after surgery, with MACEs and adverse events assessment collected at each time by clinic visits or telephone interview.

### Study endpoints

The composite primary study endpoint is the incidence of MACEs after PCI for SVGD, including death from cardiac causes, non-fatal myocardial infarction, angina pectoris, target vessel revascularization (TVR) and hospitalization for heart failure.

Secondary endpoints are defined as: (1) the occurrence of the slow/no reflow, which is defined as thrombolysis in myocardial infarction (TIMI) grade 0, 1, or 2 flow within 24 h after SVG-PCI, despite successful treatment of the vessel obstruction; (2) myocardial infarction is defined as the onset of chest pain in combination with new, typical changes in the electrocardiogram and biochemical evidence of myocardial necrosis; (3)TVR is defined as a new revascularization procedure in the target vessel; (4)successful reperfusion is defined as TIMI flow grade 3 and less than 50% residual stenosis of SVG.

### Sample size

We expect CAS of plaque and media 2.9 ± 1.7 mm^2^ among patients with MACEs and 1.9 ± 1.5 mm^2^ among patients without MACEs. It is estimated that the incidence of MACEs is 20% in 3 years after PCI for SVGD. Assuming a 20% drop-out rate, a total of 175 participants are required to provide 80% power, with a two-sided type I error of 0.05.

### Statistical analysis

Analyses will be made using SAS statistical software (version 9.3) by researchers at the Peking University Clinical Research Institute. Continuous variables will be expressed as mean ± standard deviation or median ± interquartile range (IQR), and t-test or Wilcoxon rank sum test will be used to compare the difference between groups. For categorical variables, the chi-square test/ Fisher’s exact test will be performed. Time-to-event data (MACEs in 3 year after PCI) will be presented as Kaplan-Meier estimates and the primary outcome analysis will be carried out using univariate and multivariate Cox proportional hazards regression models. The ideal cut-off of quantitative indexes of VH-IVUS and the diagnostic accuracy will be determined by the receiver-operating characteristic curve (ROC). A *p*-value < 0.05 will be considered statistically significant in all analyses.

### Trial status

The first participant was enrolled in July 2017. As of August 2018, 67 participants have been enrolled and recruitment is ongoing in Tianjin Chest Hospital, China. Treatment and follow-up of all participants are planned to continue until December 2021.

## Discussion

It has been indicated that plaque anatomy and composition identified by VH-IVUS is a predictor of long-term clinical outcomes in patients undergoing PCI. However, there have been few studies investigating the relationship between plaque composition of SVG by VH-IVUS and clinical outcome in SVGD patients undergoing PCI. PCI is a widely performed surgical procedure for SVGD patients. Considering the higher risk in conducting PCI among SVGD patients, it is necessary to explore the cardiovascular factors predicting the clinical outcome of PCI, which can be used for risk stratification and intervention guidance. In this prospective cohort study, we will identify atherosclerotic plaque components and characteristics in SVGD patients undergoing PCI and predict the long-term clinical outcomes identified with VH-IVUS.

Multiple novel intravascular imaging technologies have been developed to help identify high risk plaque characteristics in predicting cardiovascular adverse events [22, 29, 30]. Young Joon Hong et al. investigated the relationship between intravascular IVUS findings and the no-reflow phenomenon after PCI for SVGD [22]. But IVUS has limited value for identifying specific plaque components [31]. Computed tomography (CT) is routinely used for coronary angiography and can identify high risk features of plaques. Daniel R. Obaid et al. found that plaque components and classifications based on CT could not reliably classify plaques and identify TCFA [30]. With the highest spatial resolution, optical coherence tomography (OCT) has emerged as an important imaging modality for intracoronary evaluation [32]. The direct comparison between VH-IVUS and OCT by Brown et al. found that both VH-IVUS and OCT could identify advanced coronary plaques and that combined VH-IVUS/OCT was better than either alone [33]. However, OCT has a low signal penetration through lipid or necrotic core, and cannot adequately acquire images of the whole vessels with large lumen diameter or large necrotic core [29, 34]. This presents a problem for imaging of large vessels including vein grafts. The presence of macrophages, foam cells, microcalcifications, or hemosiderin, often co-existent with the necrotic core, could be adverse to accurate OCT assessment of lipidic plaque [29, 34]. VH-IVUS, which has become clinically available, can assess both plaque morphology and tissue characteristics using spectral and amplitude analysis of backscattered radiofrequency ultrasound signal. With different plaque components exhibiting a defined spectrum, VH-IVUS can classify atherosclerotic plaque into four types: FT, FF, NC, and DC [35, 36]. The radiofrequency signal is mathematically transformed into a color-coded representation, including lipid, fibrous tissue, calcification, and necrotic core [37]. Thus, VH-IVUS can identify atherosclerotic plaques exactly like histopathology.

Some meta-analysis studies have found that the relationship between absolute volume of NC components on VH-IVUS imaging and distal embolization after PCI in acute coronary syndrome (ACS) patients [35, 38]. Previous studies have demonstrated that TCFA, plaque burden, and minimum luminal area are associated with MACEs among ACS patients undergoing PCI [25, 26]. The VIVA (VH-IVUS in Vulnerable Atherosclerosis) Study showed that VH-IVUS TCFA was associated with nonrestenotic and total MACEs on individual plaque or whole patient analysis [26]. It was reported by the Providing Regional Observations to Study Predictors of Events in the Coronary Tree (PROSPECT) study that plaque burden > 70%, minimum luminal area < 4 mm^2^, and VH-IVUS TCFA were the independent predictors of nonculprit lesion–related events. However, there are limited studies investigating the associations of degenerative lesions or plaque components of SVGD with clinical outcomes using VH-IVUS. Man-Hong Jim et al. sought to report the VH-IVUS findings in degenerative aortocoronary SVG lesions and correlate plaque compositions with clinical characteristics [39]. Lesions with a plaque burden ≥70% was found to be positively correlated with FF tissue, but negatively correlated with DC [39]. However, it is a cross-sectional study, the temporary relationship between VH-IVUS findings and clinical characteristics cannot be determined. As far as the authors know, there is currently no study exploring the relationship between VH-IVUS defined atherosclerotic plaque components and clinical outcomes in SVGD patients undergoing PCI.

The current study is subject to several limitations. This is an observational cohort study, confounding effects cannot be ruled out entirely. However, the multivariate Cox proportional regression model will be used to determine the independent effects of VH-IVUS findings on long-term clinical outcomes after adjusting for the impact of baseline characteristics. Second, participants with SVGD who are enrolled in a single center (Cardiovascular Institute, Tianjin Chest Hospital) may not represent the population in China. However, we speculate that the relationship between VH-IVUS findings and clinical outcomes should persist in other SVGD patients undergoing PCI, but with varying magnitudes of association. Third, although VH-IVUS is a widely used method in clinical setting, VH-IVUS has some technological limitations. VH-IVUS TCFA is not equivalent to histopathologic definitions and VH-IVUS tend to over-estimate TCFA compared with histology.

## Conclusion

The proposed prospective cohort study aims to investigate whether plaque characteristics and components of culprit SVG lesion assessed by VH-IVUS could predict subsequent clinical outcome after PCI among SVGD patients. Once our hypothesis is confirmed, an innovative prediction tool of clinical outcomes can be created, which may lead to the development of new methods of risk stratification and intervention guidance.

## Abbreviations

ACS: Acute coronary syndrome
CABG: coronary artery bypass grafting
CAD: coronary artery disease
CAS: cross-sectional areas
CT: Computed tomography
CTFC: Corrected TIMI frame count
DC: dense calcium
EEM: external elastic membrane
FCa: fibrocalcific plaque
FF: fibrofatty
FT: fibrous tissue
IVUS: intravascular ultrasound
MACEs: major adverse cardiac events
NC: necrotic core
OCT: Optical coherence tomography
PCI: percutaneous coronary intervention
PIT: Pathologic intimal thickening
PROSPECT: Providing Regional Observations to Study Predictors of Events in the Coronary Tree
ROC: Receiver-operating characteristic curve
SVGD: saphenous vein grafts disease
SVGs: saphenous vein grafts
TCFA: Thin-cap fibroatheroma
ThCFA: Thick-cap fibroatheroma
TIMI: Thrombolysis in myocardial infarction
TLR: target lesion revascularization
TVR: target vessel revascularization
VH-IVUS: virtual histology intravascular ultrasound

## References

[1] Parang P, Arora R (2009). Coronary vein graft disease: pathogenesis and prevention. Can J Cardiol.

[2] Yayla C, Canpolat U, Akyel A, Yayla KG, Yilmaz S, Acikgoz SK, Ozcan F, Turak O, Dogan M, Yeter E (2016). Association between platelet to lymphocyte ratio and saphenous vein graft disease. Angiology.

[3] Gaudino M, Puskas JD, Di Franco A, Ohmes LB, Iannaccone M, Barbero U, Glineur D, Grau JB, Benedetto U, D'Ascenzo F (2017). Three arterial grafts improve late survival: a meta-analysis of propensity-matched studies. Circulation.

[4] Owens CD (2010). Adaptive changes in autogenous vein grafts for arterial reconstruction: clinical implications. J Vasc Surg.

[5] Barbero U, Iannaccone M, d’Ascenzo F, Barbero C, Mohamed A, Annone U, Benedetto S, Celentani D, Gagliardi M, Moretti C (2016). 64 slice-coronary computed tomography sensitivity and specificity in the evaluation of coronary artery bypass graft stenosis: a meta-analysis. Int J Cardiol.

[6] Demircelik B, Cakmak M, Nazli Y, Gurel OM, Akkaya N, Cetin M, Cetin Z, Selcoki Y, Kurtul A, Eryonucu B (2014). Adropin: a new marker for predicting late saphenous vein graft disease after coronary artery bypass grafting. Clin Invest Med.

[7] Akpinar I, Sayin MR, Gursoy YC, Karabag T, Kucuk E, Buyukuysal MC, Aydin M, Haznedaroglu IC (2014). Plateletcrit. A platelet marker associated with saphenous vein graft disease. Herz.

[8] Harskamp RE, Lopes RD, Baisden CE, de Winter RJ, Alexander JH (2013). Saphenous vein graft failure after coronary artery bypass surgery: pathophysiology, management, and future directions. Ann Surg.

[9] Levine GN, Bates ER, Blankenship JC, Bailey SR, Bittl JA, Cercek B, Chambers CE, Ellis SG, Guyton RA, Hollenberg SM (2011). 2011 ACCF/AHA/SCAI guideline for percutaneous coronary intervention: executive summary: a report of the American College of Cardiology Foundation/American Heart Association task force on practice guidelines and the Society for Cardiovascular Angiography and Interventions. Circulation.

[10] D'Ascenzo F, Barbero U, Moretti C, Palmerini T, Della Riva D, Mariani A, Omede P, DiNicolantonio JJ, Biondi-Zoccai G, Gaita F (2014). Percutaneous coronary intervention versus coronary artery bypass graft for stable angina: meta-regression of randomized trials. Contemp Clin Trials.

[11] Lee MS, Park SJ, Kandzari DE, Kirtane AJ, Fearon WF, Brilakis ES, Vermeersch P, Kim YH, Waksman R, Mehilli J (2011). Saphenous vein graft intervention. JACC Cardiovasc Interv.

[12] Yap CH, Sposato L, Akowuah E, Theodore S, Dinh DT, Shardey GC, Skillington PD, Tatoulis J, Yii M, Smith JA (2009). Contemporary results show repeat coronary artery bypass grafting remains a risk factor for operative mortality. Ann Thorac Surg.

[13] Morrison DA, Sethi G, Sacks J, Henderson WG, Grover F, Sedlis S, Esposito R (2002). Investigators of the Department of Veterans Affairs Cooperative Study AWESOME: percutaneous coronary intervention versus repeat bypass surgery for patients with medically refractory myocardial ischemia: AWESOME randomized trial and registry experience with post-CABG patients. J Am Coll Cardiol.

[14] Brilakis ES, Rao SV, Banerjee S, Goldman S, Shunk KA, Holmes DR, Honeycutt E, Roe MT (2011). Percutaneous coronary intervention in native arteries versus bypass grafts in prior coronary artery bypass grafting patients: a report from the National Cardiovascular Data Registry. JACC Cardiovasc Interv.

[15] de Vries MR, Simons KH, Jukema JW, Braun J, Quax PH (2016). Vein graft failure: from pathophysiology to clinical outcomes. Nat Rev Cardiol.

[16] Brilakis ES, O'Donnell CI, Penny W, Armstrong EJ, Tsai T, Maddox TM, Plomondon ME, Banerjee S, Rao SV, Garcia S (2016). Percutaneous coronary intervention in native coronary arteries versus bypass grafts in patients with prior coronary artery bypass graft surgery: Insights From the Veterans Affairs Clinical Assessment, Reporting, and Tracking Program. JACC Cardiovasc Interv.

[17] Roffi M, Mukherjee D, Chew DP, Bhatt DL, Cho L, Robbins MA, Ziada KM, Brennan DM, Ellis SG, Topol EJ (2002). Lack of benefit from intravenous platelet glycoprotein IIb/IIIa receptor inhibition as adjunctive treatment for percutaneous interventions of aortocoronary bypass grafts: a pooled analysis of five randomized clinical trials. Circulation.

[18] Redfors B, Genereux P, Witzenbichler B, McAndrew T, Diamond J, Huang X, Maehara A, Weisz G, Mehran R, Kirtane AJ, et al. Percutaneous coronary intervention of saphenous vein graft. Circ Cardiovasc Interv. 2017;10(5). 10.1161/CIRCINTERVENTIONS.117.004953 28495896

[19] Ohshima K, Ikeda S, Kadota H, Yamane K, Izumi N, Ohshima K, Hamada M (2013). Impact of culprit plaque volume and composition on myocardial microcirculation following primary angioplasty in patients with ST-segment elevation myocardial infarction: virtual histology intravascular ultrasound analysis. Int J Cardiol.

[20] Iijima R, Shinji H, Ikeda N, Itaya H, Makino K, Funatsu A, Yokouchi I, Komatsu H, Ito N, Nuruki H (2006). Comparison of coronary arterial finding by intravascular ultrasound in patients with "transient no-reflow" versus "reflow" during percutaneous coronary intervention in acute coronary syndrome. Am J Cardiol.

[21] Tanaka A, Kawarabayashi T, Nishibori Y, Sano T, Nishida Y, Fukuda D, Shimada K, Yoshikawa J (2002). No-reflow phenomenon and lesion morphology in patients with acute myocardial infarction. Circulation.

[22] Hong YJ, Jeong MH, Ahn Y, Kang JC, Mintz GS, Kim SW, Lee SY, Kim SY, Pichard AD, Satler LF (2012). Intravascular ultrasound findings that are predictive of no reflow after percutaneous coronary intervention for saphenous vein graft disease. Am J Cardiol.

[23] Zhao XY, Wang XF, Li L, Zhang JY, Du YY, Yao HM (2013). Plaque characteristics and serum pregnancy-associated plasma protein a levels predict the no-reflow phenomenon after percutaneous coronary intervention. J Int Med Res.

[24] Raichlin E, Bae JH, Kushwaha SS, Lennon RJ, Prasad A, Rihal CS, Lerman A (2009). Inflammatory burden of cardiac allograft coronary atherosclerotic plaque is associated with early recurrent cellular rejection and predicts a higher risk of vasculopathy progression. J Am Coll Cardiol.

[25] Yun KH, Mintz GS, Farhat N, Marso SP, Taglieri N, Verheye S, Foster MC, Margolis MP, Templin B, Xu K (2012). Relation between angiographic lesion severity, vulnerable plaque morphology and future adverse cardiac events (from the providing regional observations to study predictors of events in the coronary tree study). Am J Cardiol.

[26] Calvert PA, Obaid DR, O'Sullivan M, Shapiro LM, McNab D, Densem CG, Schofield PM, Braganza D, Clarke SC, Ray KK (2011). Association between IVUS findings and adverse outcomes in patients with coronary artery disease: the VIVA (VH-IVUS in vulnerable atherosclerosis) study. JACC Cardiovasc Imaging.

[27] Wood FO, Badhey N, Garcia B, Abdel-karim AR, Maini B, Banerjee S, Brilakis ES (2010). Analysis of saphenous vein graft lesion composition using near-infrared spectroscopy and intravascular ultrasonography with virtual histology. Atherosclerosis.

[28] Maehara A, Cristea E, Mintz GS, Lansky AJ, Dressler O, Biro S, Templin B, Virmani R, de Bruyne B, Serruys PW (2012). Definitions and methodology for the grayscale and radiofrequency intravascular ultrasound and coronary angiographic analyses. JACC Cardiovasc Imaging.

[29] Iannaccone M, Quadri G, Taha S, D'Ascenzo F, Montefusco A, Omede P, Jang IK, Niccoli G, Souteyrand G, Yundai C (2016). Prevalence and predictors of culprit plaque rupture at OCT in patients with coronary artery disease: a meta-analysis. Eur Heart J Cardiovasc Imaging.

[30] Obaid DR, Calvert PA, Gopalan D, Parker RA, Hoole SP, West NE, Goddard M, Rudd JH, Bennett MR (2013). Atherosclerotic plaque composition and classification identified by coronary computed tomography: assessment of computed tomography-generated plaque maps compared with virtual histology intravascular ultrasound and histology. Circ Cardiovasc Imaging.

[31] Pu J, Mintz GS, Brilakis ES, Banerjee S, Abdel-Karim AR, Maini B, Biro S, Lee JB, Stone GW, Weisz G (2012). In vivo characterization of coronary plaques: novel findings from comparing greyscale and virtual histology intravascular ultrasound and near-infrared spectroscopy. Eur Heart J.

[32] Niccoli G, Giubilato S, Di Vito L, Leo A, Cosentino N, Pitocco D, Marco V, Ghirlanda G, Prati F, Crea F (2013). Severity of coronary atherosclerosis in patients with a first acute coronary event: a diabetes paradox. Eur Heart J.

[33] Brown AJ, Obaid DR, Costopoulos C, Parker RA, Calvert PA, Teng Z, Hoole SP, West NE, Goddard M, Bennett MR (2015). Direct comparison of virtual-histology intravascular ultrasound and optical coherence tomography imaging for identification of thin-cap Fibroatheroma. Circ Cardiovasc Imaging.

[34] Mintz GS (2015). Optical coherence tomography and virtual-histology intravascular ultrasound: strange bedfellows? ... Or not?. Circ Cardiovasc Imaging.

[35] Ding S, Xu L, Yang F, Kong L, Zhao Y, Gao L, Wang W, Xu R, Ge H, Jiang M (2014). Association between tissue characteristics of coronary plaque and distal embolization after coronary intervention in acute coronary syndrome patients: insights from a meta-analysis of virtual histology-intravascular ultrasound studies. PLoS One.

[36] Vince DG, Dixon KJ, Cothren RM, Cornhill JF (2000). Comparison of texture analysis methods for the characterization of coronary plaques in intravascular ultrasound images. Comput Med Imaging Graph.

[37] Kawasaki M, Takatsu H, Noda T, Ito Y, Kunishima A, Arai M, Nishigaki K, Takemura G, Morita N, Minatoguchi S (2001). Noninvasive quantitative tissue characterization and two-dimensional color-coded map of human atherosclerotic lesions using ultrasound integrated backscatter: comparison between histology and integrated backscatter images. J Am Coll Cardiol.

[38] Claessen BE, Maehara A, Fahy M, Xu K, Stone GW, Mintz GS (2012). Plaque composition by intravascular ultrasound and distal embolization after percutaneous coronary intervention. JACC Cardiovasc Imaging.

[39] Jim MH, Hau WK, Ko RL, Siu CW, Ho HH, Yiu KH, Lau CP, Chow WH (2010). Virtual histology by intravascular ultrasound study on degenerative aortocoronary saphenous vein grafts. Heart Vessel.

